# Influence of Galectin-3 on the Innate Immune Response during Experimental Cryptococcosis

**DOI:** 10.3390/jof7060492

**Published:** 2021-06-20

**Authors:** Caroline Patini Rezende, Patricia Kellen Martins Oliveira Brito, Thiago Aparecido Da Silva, Andre Moreira Pessoni, Leandra Naira Zambelli Ramalho, Fausto Almeida

**Affiliations:** 1 Department of Biochemistry and Immunology, Ribeirao Preto Medical School, University of Sao Paulo, Ribeirao Preto 14049-900, SP, Brazil; ca.rezende@usp.br (C.P.R.); andrepessoni1@gmail.com (A.M.P.); 2 Department of Cellular and Molecular Biology, Ribeirao Preto Medical School, University of Sao Paulo, Ribeirao Preto 14049-900, SP, Brazil; patriciakellen04@hotmail.com (P.K.M.O.B.); sthiagoap@gmail.com (T.A.D.S.); 3 Department of Pathology, Ribeirao Preto Medical School, University of Sao Paulo, Ribeirao Preto 14049-900, SP, Brazil; lramalho@fmrp.usp.br

**Keywords:** *Cryptococcus neoformans*, galectin-3 (Gal-3), innate antifungal immunity

## Abstract

*Cryptococcus neoformans*, the causative agent of cryptococcosis, is the primary fungal pathogen that affects the immunocompromised individuals. Galectin-3 (Gal-3) is an animal lectin involved in both innate and adaptive immune responses. The present study aimed to evaluate the influence of Gal-3 on the *C. neoformans* infection. We performed histopathological and gene profile analysis of the innate antifungal immunity markers in the lungs, spleen, and brain of the wild-type (WT) and Gal-3 knockout (KO) mice during cryptococcosis. These findings suggest that Gal-3 absence does not cause significant histopathological alterations in the analyzed tissues. The expression profile of the genes related to innate antifungal immunity showed that the presence of cryptococcosis in the WT and Gal-3 KO animals, compared to their respective controls, promoted the upregulation of the pattern recognition receptor (PRR) responsive to mannose/chitin (*mrc1*) and a gene involved in inflammation (*ccr5*), as well as the downregulation of the genes related to signal transduction (*card9*, *fos*, *ikbkb*, *jun*) and PRRs (*cd209a*, *colec12*, *nptx1*). The absence of Gal-3, in fungal infection, a positively modulated gene involved in phagocytosis (*sftpd*) and negatively genes involved in signal transduction (*syk* and *myd88*), proinflammatory cytokines *il-1β* and *il-12b* and *cd209a* receptor. Therefore, our results suggest that Gal-3 may play an essential role in the development of antifungal immune responses against cryptococcosis.

## 1. Introduction

*Cryptococcus neoformans*, the primary etiological agent of cryptococcosis, is a fungal pathogen that affects the immunocompromised individuals [1]. Yeast is found in several environmental niches and is generally associated with avian guano or vegetation [2]. After inhalation of yeast or desiccated basidiospores [3], this pathogen can spread from the lungs to the central nervous system, especially if the cellular immunity is compromised [4,5]. The main virulence factors of this pathogen include its polysaccharide capsule, the capacity to grow at 37 °C, and the melanin deposition on its cell wall [6].

Galectins are a family of highly conserved animal lectins with a common carbohydrate recognition domain (CRD) capable of binding to β-galactosides [7]. Members of this family are present in vertebrates, invertebrates, and protists [8]. Galectins bind to the exogenous glycans exposed on the surface of viruses, bacteria, fungi, and parasites, thereby revealing their essential role in the recognition of pathogens by innate immune system [9].

Galectin-3 (Gal-3), the most studied member of the galectin family, is expressed in the nucleus, cytoplasm, mitochondria, cell surface, and extracellular space [10]. Its biological functions are defined by its cellular localization and it plays important roles in the cell-cell or cell-matrix interactions, cell growth and differentiation, angiogenesis, and apoptosis [11]. Gal-3 may contribute to the innate immune response via pathogen recognition, regulatory cytokine production, cell migration from the innate immune system to the inflammation site, recruitment, and improvement in the macrophage survival and antimicrobial activities [12]. In addition, the analysis of the expression levels of the genes associated with innate immunity in Gal-3 KO mice under physiological conditions showed the importance of Gal-3 in the innate immune response of the host [13].

Some studies have suggested important roles of Gal-3 in the fungal infections caused by *Aspergillus fumigatus* [14], *Candida albicans* [15], *Cryptococcus neoformans* [16], *Histoplasma capsulatum* [17], and *Paracoccidioides brasiliensis* [18,19]. This study investigated the Gal-3 absence effect on the gene profile of the innate immunity of the host during experimental cryptococcosis. The gene profile analysis of innate immunity in the lungs, spleen, and brain during infection by *C. neoformans*, suggests that Gal-3 is involved in the upregulation of PRRs responsive to β-glucan and mannose/chitin (*clec7a* and *mrc1*), as well as the genes related to inflammation (*cxcl1*, *cxcl9*, *cxcl10*, *ccr1*, *ccr5*, *il-6*), and phagocytosis (*sftpd*). On the other hand, it is also involved in the downregulation of *cd209a* receptor, proinflammatory cytokines *il-1β* and *il-12b*, and the genes involved in signal transduction (*syk* and *myd88*).

## 2. Materials and Methods

### 2.1. Animals

We used male C57BL/6 (wild type, WT) and Gal-3 knockout (Gal-3 KO) mice at six to eight weeks of age. Gal3 -/- mice were previously generated as described and bred on a C57BL/6 mouse background for nine generations [20]. The animals were housed in the animal facility of the Ribeirao Preto Medical School, University of São Paulo, Ribeirao Preto, Brazil under optimized hygienic conditions. All animal experiments were conducted following the Brazilian College of Animal Experimentation, Brasilia, Brazil and were approved by the Committee on Ethics in Animal Research of the Ribeirao Preto Medical School at the University of Sao Paulo, Protocol 100/2015 (27 January 2016).

### 2.2. Cryptococcus Neoformans Infection

The *C. neoformans* strain H99 (Serotype A) yeast cells were grown for 24 h at 30 °C with constant shaking at 150 rpm in a Sabouraud dextrose broth, harvested by centrifugation at 7500× *g* for 10 min at 25 °C, washed in sterile phosphate-buffered saline (PBS), and counted using the Indian ink in a Neubauer chamber. The C57BL/6 mice were anesthetized with 2% isoflurane using a rodent anesthesia device. Then, their necks were hyperextended and the trachea was exposed after incision. The inoculum (10^6^ cells/mL) suspended in 50 µL of PBS was inoculated intratracheally using a 0.5 mL syringe and 30 gauge needle. The incision was later sutured using 4-0 silk sutures. The control group received intratracheal PBS. The inoculum concentration was confirmed by plating on the Sabouraud dextrose agar.

### 2.3. Histopathological Analysis

Fragments of the lungs, spleens, and brains from the WT and Gal-3 KO mice, control and infected with *C. neoformans*, were processed for histopathological analysis. The tissues were fixed in buffered formalin, dehydrated in a series of ethyl alcohol, diaphanized in xylol, and embedded in paraffin. The serial sections were 5-μm thick and stained with hematoxylin and eosin (H&E). The sections were then analyzed using an Olympus microscope at 20× magnification. Five slides per organ were analyzed for each control and experimental group.

### 2.4. Polymerase Chain Reaction (PCR) Array

The PCR Array was performed as previously described by Rezende et al. [13]. WT and Gal-3 KO mice were euthanized, seven days post-infection, and the lungs, spleens, and brain fragments were aseptically harvested and homogenized in a tissue homogenizer (IKA^®^ T10 basic homogenizer, Campinas, SP, Brazil). Total RNA was extracted from the lungs, spleen, and brain homogenates using the TRIzol reagent (Invitrogen Corporation, Carlsbad, CA, USA), according to the manufacturer’s instructions. The concentration of the eluted total RNA was assessed by measuring the absorbance ratios (A260/A280 and A260/A230) on a spectrophotometer (NanoDrop, Thermo Fisher Scientific-Wilmington, DE, USA). RNA integrity was evaluated by electrophoresis on 1.8% agarose gel to check for the presence of the 28S and 18S bands. Reverse transcription into cDNA from oligo d(T) primers was performed using the ImProm-II^TM^ Reverse Transcription System Kit (Promega Corp., Fitchburg, WI, USA). The Mouse Antifungal Response RT² Profiler PCR Array Kit (Qiagen- Cat #PAMM-147Z) was used for the gene expression analysis. This arrangement consisted of a plate containing 84 lyophilized primers targeting the genes involved in the innate immune response to fungal infection (cited below), negative controls of genomic DNA and primers for six different constituent genes. A pool of cDNA was added to a solution containing the buffer, DNA polymerase, SYBR green Mastermix (2X RT^2^ SYBR Green Mastermix, SABiosciences-Qiagen, Hilden, NW, Germany), and water. This solution was partitioned among the 96 wells of the plate (25 μL per well). Real-time PCR was performed in a BioRad CFX 96 thermal cycler (C1000^TM^ thermal cycler, Hercules, CA, USA) with the following conditions: 95 °C for 10 min followed by 40 cycles at 95 °C for 15 s and 60 °C for 60 s. The threshold cycle (Ct) values were analyzed using the SABiosciences Web software available online via the Qiagen Data Analysis Center website (http://www.qiagen.com/geneglobe; accessed on 19 February 2019).

### 2.5. Genes Analyzed Using the Mouse Antifungal Response RT² Profiler PCR Array Kit (Qiagen Cat# PAMM-147Z)

Table 1 shows genes analysis by using the Mouse Antifungal Response RT^2^ Profiler PCR Array Kit.

### 2.6. Statistical Analysis

Statistical analysis was performed as described by Rezende et al. [13] with slight modifications. The Ct values were normalized using the selection method (*gusb*, *hprt1*, *hsp90ab1*, *gadph* and *actb*), which showed more stable expression between the experimental and control groups. If more than one control gene was selected for normalization, the geometric mean of its values can be determined. Our data were normalized with *grapdh*, *gusb* and *b2m* for the lungs; *hsp90ab1* and *actb* for the spleen, and *actb*, *gusb* and *hsp90ab1* for the brain. The Ct values were geometrically calculated and used to determine the 2^−ΔΔCt^. Differences in the transcript levels (fold change [ FC]) between the experimental and control groups were determined using the Ct comparison method, based on the 2^−ΔΔCt^ algorithm. Genes were considered significantly modulated (induced or repressed) if the difference in the mean 2^−ΔΔCt^ values was more significant than 2 or less than −2.

## 3. Results

In this section, we will discuss the results from the histopathological analysis and changes in the expression of antifungal immunity genes in the lung, spleen and brain of WT and Gal-3 KO animals in the presence and absence of cryptococcosis.

### 3.1. Histopathological Analysis of WT and Gal-3 KO Mice during C. neoformans Infection

**Figure 1:**
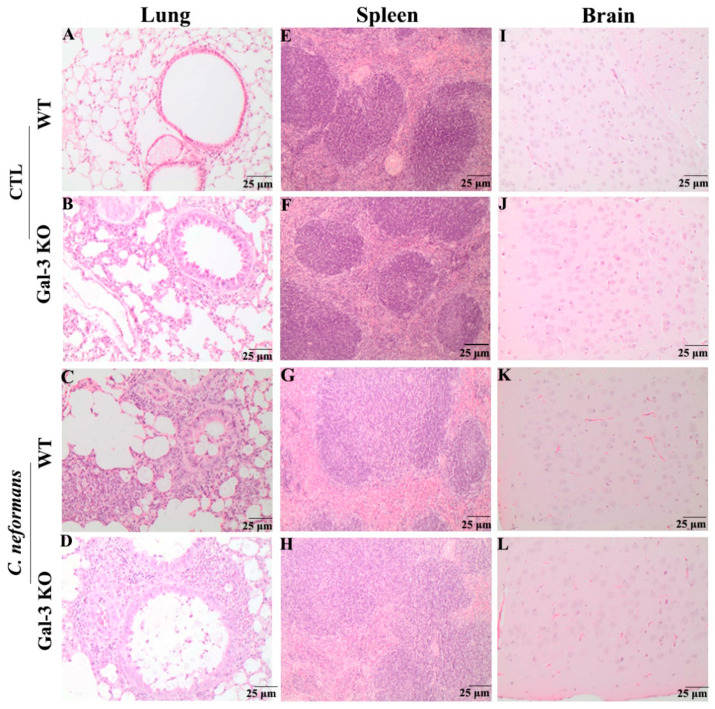
Histopathology of the lungs, spleens, and brains of the wild-type (WT) and Gal-3 knockout (KO) mice during the *C. neoformans* infection. Mice were intratracheally infected with 10^6^ yeast cells or received PBS (control group-CTL), and their organs were analyzed seven days post-infection. The images represent the organs sections from WT and Gal-3 KO mice in the presence or absence of *C. neoformans* infection. In lungs, the absence of infection promoted higher mucus secretion in Gal-3 KO mice compared to the WT mice (**A**,**B**), whereas in cryptococcosis there was moderate peribronchial eosinophilia in both the WT and Gal-3 KO animals (**C**,**D**). Significant alterations were not detected in the spleens and brain (**E**–**L**). Tissue sections were stained with hematoxylin/eosin and the images were captured with an Olympus microscope equipped with a photo-documentation system. Scale bar = 25 µm for all organs. The slides shown are representative of ten samples per treatment group.

To evaluate whether inflammatory infiltrates are promoted by the absence of Gal-3, we performed a histopathological analysis of the lungs, spleen, and brain from WT and Gal-3 KO mice during *C. neoformans* infection. In the absence of infection, we observed higher mucus secretion by the bronchial epithelium in the lungs of Gal-3 KO when compared to WT mice. Still, this alteration was not significant (Figure 1A,B). During *C. neoformans* infection, was observed moderate peribronchial eosinophilia in the lungs of WT animals similar to Gal-3 KO animals (Figure 1C,D). After seven days post-infection, it is possible to observe an increase in the fungal burden in the spleen and brain [16]. However, we did not detect significant alterations in the spleen and brain, in either the absence or presence of infection (Figure 1E–L). These findings suggest that Gal-3 absence does not cause significant histopathological alterations in the analyzed tissues.

### 3.2. Experimental Cryptococcosis Influence the Gene Expression Related to Inflammation, Signal Transduction Pathways and Fungal Standard Recognition Receptors

*C. neoformans* infection promoted an increase in the fungal burden in the lung, spleen and brain of Gal-3 KO mice, seven days post-infection [16]. However, further studies are needed to understand the Gal-3 involvement in the innate immune response during cryptococcosis. We analyzed the expression levels of 84 genes in WT and Gal-3 KO mice compared to their respective controls and Gal-3 KO mice relative to WT mice, at seven days post-infection (Table S1).

**Figure 2:**
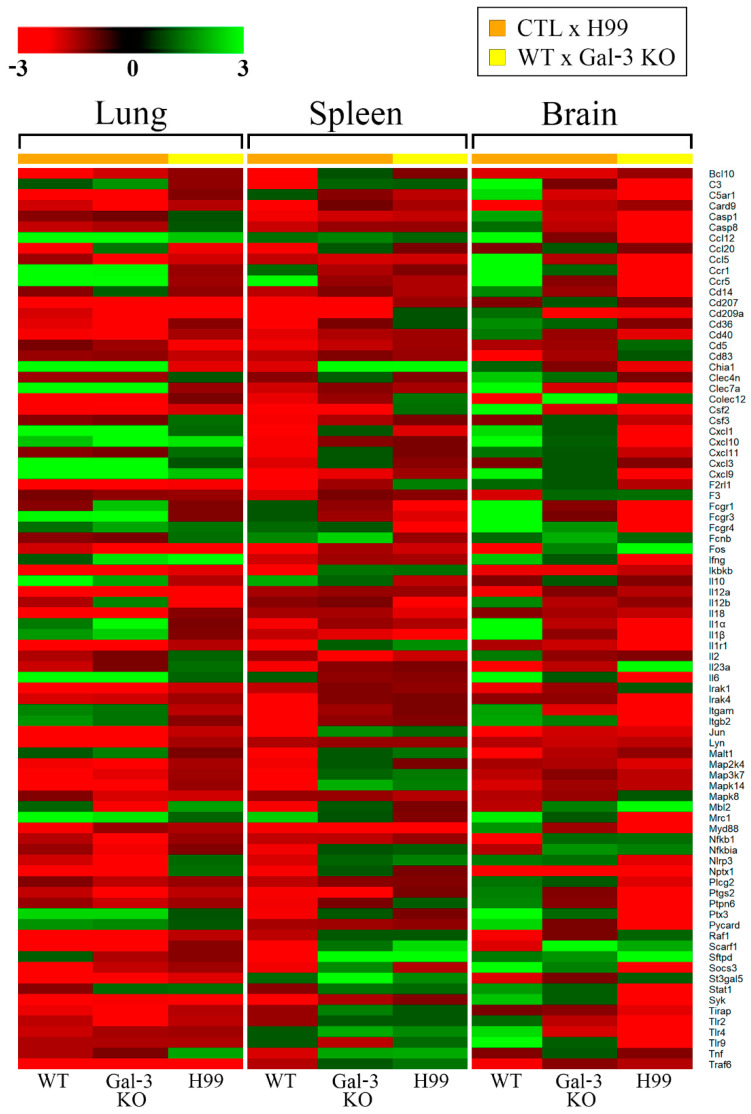
Heat map of gene expression of innate antifungal immunity in the lungs, spleen, and brain of the WT and Gal-3 KO mice during cryptococcosis. The analyses were based on the following comparisons: *C. neoformans* infection compared to the control group, in both the WT and Gal-3 KO animals (represented by orange bars), and WT compared to Gal-3 KO animals infected situation (represented by yellow bars). The analyses were performed in the lungs, spleens, and brains after seven days of *C. neoformans* infection. All control animals received phosphate-buffered saline (PBS) intratracheally. The scale represents the fold change, with a threshold equal to three (green: upregulated, red; downregulated, black: medium change).

When analyzing WT and Gal-3 KO mice compared to their respective controls (WT and Gal-3 KO CTL), a total of 18 and 40 genes were upregulated and downregulated in the lungs, respectively (Table S2). We observed that experimental *C. neoformans* infection promoted overexpression of 14 and 17 genes involved in innate immunity, in the presence and absence of Gal-3, respectively. In addition, we observed that *il-10* was exclusively modulated in the infected WT mice, whereas the absence of Gal-3 during infection promoted overexpression of *fcgr1*, *ifn-ɣ*, *il-1α*, and *il-1β*, as compared to uninfected mice. Under the same conditions, 21 genes were downregulated in the lungs of WT and Gal-3 KO mice during cryptococcosis. The expression of *bcl10*, *ccl20*, *map3k7*, *myd88*, and *socs3* was exclusive to the infected WT mice, whereas in the absence of Gal-3 promoted expression decreased of *card9*, *ccl5*, *cd36*, *cd209a*, *fos*, *mbl2*, *nfkb1*, *nfkbia*, *nlrp3*, *ptgs2*, *tirap*, and *tlr2* (Figure 2).

In the spleen, 8 and 44 genes presented up and downregulation, respectively (Table S3). During infection by *C. neoformans*, mannose receptor C-type 1 (*mrc1*) was upregulated in both WT and Gal-3 KO mice compared to the control group. WT mice also showed significantly increased expression of the chemokine C-C motif receptor (*ccr5*) and mitogen-activated Protein Kinase 14 (*mapk14*). In contrast, in the infected Gal-3 KO mice, we observed upregulation of *chia1*, *fcnb*, *sftpd*, and *st3gal5*. Additionally, in the downregulated group, PRRs responsive to mannose/chitin (*cd207*, *cd209a*) and inflammation (*csf2*, *myd88*, *ptgs2*) were commonly regulated in WT and Gal-3 KO mice with cryptococcosis (Figure 2).

Among the three organs analyzed, the brain showed major changes in gene expression related to the antifungal innate immune response. In total, 33 and 53 genes were upregulated and downregulated, respectively (Table S4). Infected WT and Gal-3 KO mice, compared to their control groups, presented 26 and 3 genes upregulated, respectively, which are involved in inflammation (*c3*, *ccl5*, *ccl12*, *ccr1*, *ccr5*, *csf2*, *cxcl1*, *cxcl9*, *cxcl10*, *ifn-γ*, *il-1α*, *il-1β*, *il-6*), phagocytosis (*colec12*, *fcgr1*, *fcgr3*, *fcgr4*, *fcnb*), signal transduction via Dectin-1(*syk*), NOD-like receptors (*pycard*), complement system *(c5ar1*), fungal standard recognition receptors (*clec4n*, *clec7a*, *mcr1*, *ptx3*, *scarf1*, *tlr4*, *tlr9*), and gene responsive to pathogenic fungi (*socs3*). Concerning downregulated genes, the gene encoding neuronal pentraxin 1 (*nptx1*) was downregulated in both groups. In addition, the others 13 genes (*card9*, *cd83*, *colec12*, *fos*, *il-12a*, *il-23a*, *ikbkb*, *irak1*, *jun*, *malt1*, *nfkb1*, *raf1*, *traf6*) had lower expression in WT mice and *cd209a* in Gal-3 KO mice (Figure 2).

**Figure 3:**
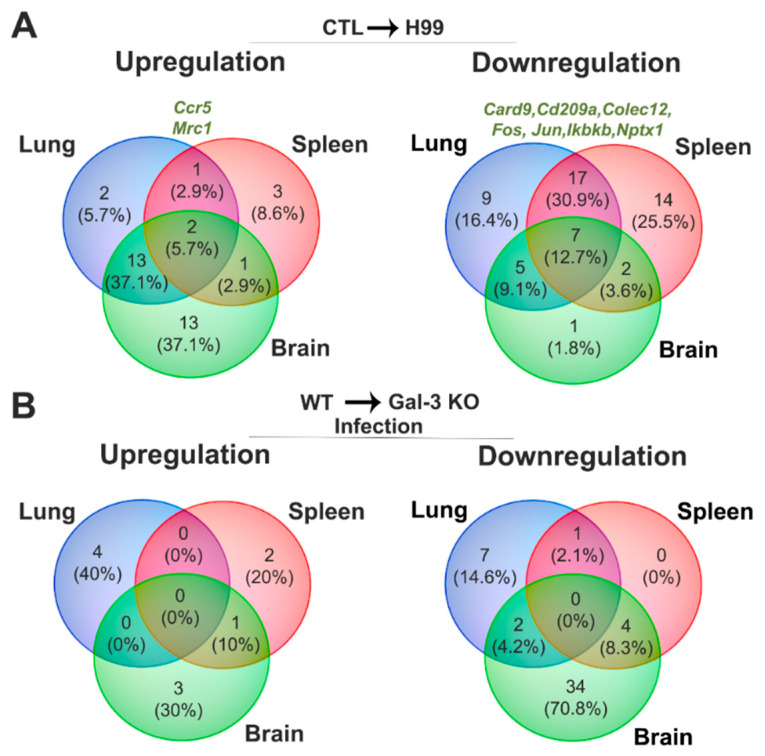
Expression levels of the genes involved in the mouse antifungal response in the absence of Gal-3 during cryptococcosis. Comparative Venn diagram of expressed genes up and downregulated in the lungs, spleen, and brain, during experimental cryptococcosis (H99) compared to the control group (CTL) in WT and Gal-3 KO animals (WT CTL vs. WT H99 and Gal-3 KO CTL vs. Gal-3 KO H99) (**A**) and absence of Gal-3 related to WT mice in *C. neoformans* infection (WT H99 vs. Gal-3 KO H99) (**B**). Venn diagram was designed using Venny v2.1.

These results suggest that fungal infection by *C. neoformans* in WT and Gal-3 KO mice relative to their control groups promotes upregulation of mannose receptor, C, type1 (*mrc1*) and chemokine (C-C motif) receptor 5 (*ccr5*) and downregulation of *card9*, *cd209a*, *colec12*, *fos*, *ikbkb*, *jun*, and *nptx1* in the three analyzed organs (Figure 3A).

### 3.3. Gal-3 Absence Promotes Downregulation of Genes Related to Inflammation, Signal Transduction Pathways and Phagocytose during C. neoformans Infection

Our group demonstrated that the absence of Gal-3, under physiological conditions, promotes changes in the expression of genes involved in innate host immunity [13], however, we proposed to study the influence of Gal-3 on gene expression in fungal infections compared to the control animal (Gal-3 KO vs. WT). During experimental *C. neoformans* infection, Gal-3 absence promotes upregulation of chemokines (*ccl12*, *cxcl9* and *cxcl10*) and *ifn-ɣ* in the lungs (Table S2 and Figure 2). Regardless of the spleen and brain, there was a common increase in the surfactant-associated Protein D (*sftpd*) expression. Additionally, we observed the modulation of PRRs (*chia1* and *scarf1*) in the spleen (Table S3). In contrast, the genes involved in signal transduction via Toll-like receptors (*fos*) and inflammation (*il-23a* and *mbl2*) were observed in the brain (Table S4 and Figure 2). Regarding downregulation, we observed that Gal-3 absence in cryptococcosis promoted significant changes in the expression of 10, 5 and 40 genes in the lungs, spleen and brain, respectively. The lungs promoted downregulation of *ccl20*, *cd5*, *cd207*, *cd209a*, *f2rl1*, *fos*, *il-12a*, *il-12b*, *syk* and *traf6* (Table S2), while the spleen negatively modulated *fcgr1*, *fcgr4*, *il-1β*, *il-12b*, and *myd88* (Table S3). PRRs (*cd209a*, *chia1*, *clec7a*, *itgam*, *mcr1*, *nptx1*, *ptx3*, *tlr2*, *tlr4*, *tlr9)*, genes involved in signal transduction (*casp1*, *casp8*, *cd14*, *c5ar1*, *irak4*, *myd88*, *pycard*, *stat1*, *syk*), phagocytosis (*fcgr1*, *fcgr3*, *fcgr4*), inflammation (*c3*, *ccl5*, *ccl12*, *ccr1*, *ccr5*, *csf2*, *cxcl1*, *cxcl9*, *cxcl10*, *ifn-γ*, *il-1α*, *il-1β*, *il1r1*, *il-6*, *itgb2*, *ptgs2*) and genes responsive to pathogenic fungi (*socs3*, *ptpn6*) had decreased expression in the brain (Table S4 and Figure 2). 

Taken together, these results suggest that Gal-3 absence during *C. neoformans* infection promoted *sftpd*, which involved in phagocytosis, upregulation in the spleen and brain. Furthermore, the lungs and spleen showed downregulation of proinflammatory cytokine (*il-12b*). In contrast, the lungs and brain showed decreased expression of PRR responsive mannose and chitin (*cd209a*) and tyrosine kinase (*syk*) involved in signal transduction via Dectin-1 and the complement system in the spleen. In addition, the spleen and brain negatively modulated genes involved in phagocytosis (*fcgr1*, *fcgr4*) and inflammation (*il-1β*, *myd88*) (Figure 3B).

## 4. Discussion

Galectin-3 plays an important role in the expression of genes related to innate immunity in the host. Its absence, under physiological conditions, promotes differentially modulated genes involved in the transcription of beta-glucan, mannose, and chitin-responsive pattern recognition receptors, signal transduction, inflammation, and phagocytosis [13]. Recent studies have shown that Gal-3 plays important roles in inflammatory processes and immunity during fungal infections [14,15,16,17,18,19]. Thus, our objective was to evaluate the influence of Gal-3 on the host’s innate immune response to *C. neoformans* infection.

Immune host cells may promote phagocytosis of pathogens through interaction with PRRs-PAMPs [21,22]. C-type lectin receptors (CLRs), which are classes of PRRs, are important for fungal recognition and shaping the innate and adaptive immune responses [23]. Our results demonstrated that the mannose receptor (MR), an important CLR, was upregulated in the three organs analyzed during experimental cryptococcosis. This receptor is responsible for recognizing various ligands, including fungal pathogens, and participating in effective immune response [24]. Several studies have shown MR with increased expression levels after infection by Pseudomonas and HIV [25,26], and corneal epithelial cells during *Aspergillus fumigatus* infection [24]. Additionally, MR may be crucial in inducing Il-17 production in *C. albicans* [27] and has an important synergistic action with Tlr-4 and Dectin-1 in shaping the Th17 antifungal response during *P. brasiliensis* infection [28].

The clearance of *C. neoformans* infection requires Th1 immunity with subsequent pulmonary recruitment and leukocyte activation, with macrophages being the primary cell type in defense [29]. Chemokines play an important role in leukocyte recruitment, migration, and polarization of the Th1/Th2 immune response [30]. Previous studies have demonstrated that Th1 cells preferentially express Ccr5 and Cxcr3 chemokine receptors, which allow migration of these cells to the site of infection [31], corroborating our results showing upregulation of *cxcl9*, *cxcl10* in the lungs; *ccr5* in the lungs and spleen of infected WT and Gal-3 KO animals and in the brain of WT animals with cryptococcosis. In addition, in vivo studies demonstrated increased expression of *cxcr3* and its *cxcl9* and *cxcl10* ligands, and *ccr5* in the lungs of mice infected with *C. neoformans*, positively correlating with the migration pattern of Th1 cells [32,33]. Souto et al. evaluated chemokines and receptor expression and their regulation by *Ifn-ɣ* in *P. brasiliensis* infection, which showed increased production of *ccl2*, *ccl3*, *cxcl9*, *cxcl10*, *ccr5*, and *cxcr3* receptors in the lungs of infected mice with *C. neoformans* [34]. Increased *ifn-ɣ* production probably increases *cxcl9* and *cxcl10*, resulting in Th1 cell migration to the inflammatory focus [34].

The Gal-3 absence in the lungs, spleen, and brain, during *C. neoformans* infection promoted the downregulation of *cd209a*, *fcgr1*, *fgr4*, *il-1β*, *il-12b*, *myd88*, and *syk*. Recently, our group demonstrated that Gal-3 absence promoted increased fungal burden in the lungs and brain of Gal-3 KO mice by *C. neoformans* [16]. This increased susceptibility to the *C. neoformans* infection in the absence of Gal-3 has also been reported in the infections caused by other fungal pathogens, such as *A. fumigatus* [14], *C. albicans* [15], and *P. brasiliensis* [18]. These studies suggest that Gal-3 deficiency is associated with at higher spread of fungal infection and impaired Th1 and/or Th17 antifungal immune response, corroborating our detected downregulated expression of the genes for proinflammatory cytokines, *il-1β* in the spleen and brain, *il-12a* in the lungs, and *il-12b* in the lungs and spleen. Furthermore, the downregulation of *il-12* corroborates the low concentration of this cytokine in the spleen of Gal-3 KO mice after three and seven days of infection with *C. neoformans* [16].

The Gal-3 absence promotes downregulation of *cd209a*, also known as DC-SIGN, and proteins involved in its transduction pathway, such as *myd88* and *syk*. The receptor Cd209a is highly expressed in dendritic cells (DCs) and is recognized by glycans rich in mannose [35]. This recognition leads to uptake and antigen processing for subsequent presentation via MHC complex [35]. In murine macrophages infected with *C. albicans*, this receptor, together with Dectin-1 and Tlr-2, induces an oxidative burst and TNF-α production in vitro [36,37], whereas in *C. neoformans* infection, together with MR, they play a role in the recognition of mannoproteins present in the pathogen capsule [38]. Concerning the proteins involved in the transduction pathway for CLRs, it was verified that Dectin-1-Syk-Card9 signaling induced the production of proinflammatory cytokines such as Il-6, Il-12, Il-23, and TNF-α, along with the maturation of DCs and their differentiation for Th17 profile [39]. Thus, signaling pathways related to Syk-Card9 may promote TLR-independent DC activation and regulateTh17 response in some infections [39]. In agreement with these results, our group previously demonstrated that Gal-3 absence affects the Th17 immune response [16], which could be explained by the *cd209a* receptor, *myd88*, and *syk* downregulation.

Studies have shown the involvement of IgG antibodies in protection against pathogens, such as viruses, bacteria, fungi and parasites through direct or indirect effector mechanisms [40]. The main functions mediated by antibodies (Abs) are toxin neutralization, opsonization, phagocytosis, activation of the complement system, induction of reactive oxygen species (ROS), and reactive nitrogen species (RNS) [40]. The interaction of Abs with Fc receptors (FcRs) promotes additional functions such as cellular activation, cytokine production, receptor-mediated endocytosis, and degradation of immune complexes [40]. The Abs confer protection against fungal pathogens such as *C. neoformans* [41] and *C. albicans* [42]. In cryptococcosis, IgG-mediated opsonization prevents fungal growth in macrophages and promotes increased phagocytosis of the fungus [43], which is strictly dependent on FcR [43]. In our results, *fcgr1* and *fcgr4* receptors were downregulated in the spleen and brain of Gal-3 KO mice during cryptococcosis, and the downregulation of these receptors may lead to an impaired immune response and higher vulnerability to the fungal infection. Thus, our results show that the absence of Gal-3 in cryptococcosis promotes the negative modulation of genes involved in fungal recognition.

## 5. Conclusions

In conclusion, our findings suggest that during the experimental *C. neoformans* infection, Gal-3 is involved in the upregulation of the PRR response to mannose/chitin (*mrc1*) and the genes related to inflammation (*cxcl1*, *cxcl9*, *cxcl10*, *ccr1*, *ccr5*, *il-6*), and phagocytosis (*sftpd*). It is also involved in the downregulation of *cd209a* and Fc receptors, proinflammatory cytokines *il-1β* and *il-12b*, as well as the genes involved in signal transduction, such as tyrosine kinase (*syk*) and *myd88*. Therefore, we hypothesized that Gal-3 might play an important role in innate immunity in the development of effective antifungal immune response against cryptococcosis and may potentially influence the adaptive immune response as well.

## Figures and Tables

**Table 1:** RT² Profiler PCR Array Mouse Antifungal Response (Qiagen Cat# PAMM-147Z).

Fungal Pattern Recognition Receptors (PRRs)	
Responsive to beta-glucan	*cd5*, *cd36*, *clec7a*, *itgam*, *itgb2*, *scarf1*, *tlr2*
Responsive to mannose/chitin	*cd207*, *cd209a*, *chia1*, *clec4n*, *mcr1. tlr2*, *tlr4*
Others fungal PRRs	*colec12*, *nlrp3*, *nptx1*, *ptx3*, *tlr9*
**Signal Transduction**	
Dectin-1	*bcl10*, *card9*, *clec7a*, *malt1*, *plcg2*, *raf1*, *syk*
NFΚβ signaling	*bcl10*, *card9*, *casp1*, *casp8*, *cd40*, *ikkβ*, *il-10*, *il-1β*, *irak4*, *malt1*, *map3k7*, *nfkb1*, *nfkbia*, *stat1*, *tnf*
Toll-like receptor (TLR)	*casp8*, *cd14*, *fos*, *irak1*, *itgb2*, *jun*, *map2k4*, *map3k7*, *mapk14*, *mapk8*, *nfkβ*, *tirap*, *tlr2*, *tlr4*, *tlr9*
Complement signaling	*c3*, *c5ar1*, *itgam*, *itgb2*, *mbl2*, *syk*
NOD-like receptor (NLR)	*card9*, *casp1*, *nlrp3*, *pycard*
**Inflammation**	
*c3*, *ccl12*, *ccl20*, *ccl5*, *ccr1*, *ccr5*, *cd14*, *cd40*, *clec7a*, *cxcl1*, *cxcl10*, *cxcl11*, *cxcl3*, *cxcl9*, *f3*, *fos*, *il-10*, *il-1α*, *il-1β*, *il-2*, *il-23a*, *il-6*, *itgb2*, *lyn*, *mbl2*, *myd88*, *nfkb1*, *ptgs2*, *tirap*, *tlr2*, *tlr4*, *tlr9*, *tnf*	
**Phagocytosis**	
*c3*, *cd14*, *cd36*, *clec7a*, *colec12*, *fcgr1*, *fcnb*, *mbl2*, *sftpd*	
**Genes Responsive to Pathogenic Fungi**	
*Candida albicans*	*ccl12*, *ccl20*, *ccl5*, *ccr1*, *cd14*, *cd209a*, *cd83*, *clec4n*, *clec7a*, *csf2*, *csf3*, *cxcl1*, *cxcl10*, *cxcl11*, *cxcl3*, *cxcl9*, *fcgr1*, *fcgr4*, *ifng*, *il-12b*, *il-18*, *il-1α*, *il-1β*, *il1r*, *il-13a*, *il-6*, *myd88*, *nfkb1*, *ptgs2*, *socs3*, *tlr2*, *tnf*
*Aspergillus* *fumigatus*	*c3*, *casp1*, *casp8*, *ccl12*, *ccl20*, *ccl5*, *ccr1*, *ccr5*, *cd14*, *cd40*, *cd83*, *clec4n*, *clec7a*, *csf2*, *csf3*, *cxcl1*, *cxcl10*, *cxcl11*, *cxcl3*, *cxcl9*, *f2rl1*, *f3*, *fcgr1*, *fcgr3*, *fcnb*, *ifn-γ*, *il-10*, *il-12a*, *il-12b*, *il-18*, *il-1α*, *il-1β*, *il1r1*, *il-6*, *jun*, *malt1*, *mapk14*, *mbl2*, *myd88*, *nfkbia*, *ptgs2*, *ptpn6*, *ptx3*, *sftpd*, *socs3*, *st3gal5*, *stat1*, *syk*, *tlr2*, *tlr4*, *tlr9*, *tnf*
*Cryptococcus* *neoformans*	*c3*, *cd5*, *ccl12*, *cd40*, *socs3*

## Data Availability

The data used to support the findings of this study are included in this article.

## Supplementary Materials

The following are available online at https://www.mdpi.com/article/10.3390/jof7060492/s1, Table S1: Fold Regulation RT² Profiler™ PCR Array Mouse antifungal response. Table S2: gene expression profiles in the lungs of the WT and Gal-3 KO mice in the presence of *Cryptococcus neoformans* infection. Table S3: gene expression profiles in the spleens of the WT and Gal-3 KO mice in the presence of *Cryptococcus neoformans* infection. Table S4: gene expression profiles in the brains of the WT and Gal-3 KO mice in the presence of *Cryptococcus neoformans* infection.
